# Prognostic values of clinicopathological characteristics and survival outcomes in prostate infiltrating ductal carcinoma: a population-based study

**DOI:** 10.18632/oncotarget.16070

**Published:** 2017-03-10

**Authors:** Yu-Peng Wu, Shao-Hao Chen, Shi-Tao Wang, Xiao-Dong Li, Hai Cai, Yun-Zhi Lin, Xue-Yi Xue, Yong Wei, Qing-Shui Zheng, Ning Xu

**Affiliations:** ^1^ Department of Urology, First Affiliated Hospital of Fujian Medical University, Fuzhou 350005, China

**Keywords:** infiltrating ductal carcinoma, acinar cell carcinoma, PCa, SEER

## Abstract

Infiltrating ductal carcinoma (IDC) is a rare histologic subtype of prostate cancer. We investigated the clinicopathological features and prognosis of IDC compared with acinar cell carcinoma (ACC). We identified 3814 men diagnosed with prostate cancer between 2004 to and 2013 in the Surveillance, Epidemiology, and End Results database, including 511 IDC and 3303 ACC cases. Prostate cancer-specific survival (PCSS) was compared using univariate and multivariate Cox proportional hazards models. Generally, IDC occurred in older men (≥ 65 years old) and presented with larger sizes, and higher grades, American Joint Committee on Cancer (AJCC) stages, AJCC T stages, lymph node positive rates and metastasis rates. Men with IDC were less likely to undergo radical prostatectomy, but more likely to be treated with adjuvant radiation than men with ACC. Five-year PCSS rates were significantly worse in IDC. In the multivariate analysis, patients with ACC had a better PCSS than patients with IDC. In conclusion, IDC has unique clinicopathological characteristics and has worse prognosis than ACC. Multivariable Cox regression analysis showed that age over 85 years, higher grade and T stage, and metastasis at diagnosis were independent prognostic factors of worse survival outcomes, whereas radical prostatectomy was an independent prognostic factor of better survival outcomes.

## INTRODUCTION

Infiltrating ductal carcinoma (IDC) is a rare subtype of prostate cancer (PCa) and is commonly mixed with acinar elements [1]. The overall incidence of IDC is low, with an incidence ranging from 0.5% to 6% of all diagnosed PCa cases [2, 3]. Previous studies have revealed some of the characteristic properties of IDC. Although its rarity has prevented researchers from firmly defining the prognostic features of IDC, the data have suggested a less favorable clinical outcome for these cases compared with acinar cell carcinoma (ACC) cases [4, 5].

Because of its low incidence, most of the available studies are case reports or retrospective studies. Given the limited available data, a comprehensive summarization of the clinicopathological characteristics and prognostic factors associated with IDC is lacking. Therefore, theprognostic values of demographic and clinicopathological characteristics in IDC remain unclear. Previous studies [4, 5] have often lacked a detailed description of the clinical characteristics of IDC. Further, most studies lacked adjustments for confounding factors. A better understanding of the prognostic factors of IDC is needed as this information will be useful for physicians to make better therapeutic decisions. Thus, it is significant to explore the clinicopathological demographics and prognostic factors of IDC in a large population. This study used data extracted from the Surveillance, Epidemiology, and End Results (SEER) database to determine and compare survival outcomes in patients with IDC and ACC. We sought to determine the prognostic factors that may contribute to survival differences between these two histologic subtypes of PCa.

## RESULTS

### Clinicopathological characteristics of IDC

Finally, 3814 patients with PCa were enrolled, including 511 and 3303 patients with IDC and ACC, respectively. The demographics and treatment characteristics of patients with IDC were compared to those with ACC, and the results are summarized in Table 1. There were considerable differences in tumor characteristics between the arms in terms of tumor grade, tumor size, American Joint Committee on Cancer (AJCC) stage, AJCC T stage, surgery type, radiation, lymph node (LN) status, and metastasis at diagnosis. Patients with IDC had higher grade (grade III and IV: 71.6% vs. 58.2%, respectively; P< 0.001) and larger tumor size (tumor size 2–5: 11.0% vs. 5.2%, respectively; P < 0.001 and tumor size > 5: 1.2% vs. 0.5%, respectively; P < 0.001) than patients with ACC. Further, AJCC stages III and IV were more frequent among patients with IDC than among patients with ACC (20.4% vs. 14.2%, respectively; P < 0.001 and 22.3% vs. 4.0%, respectively; P < 0.001). Patients with IDC presented with more AJCC T3 and T4 stages than patients with ACC (22.5% vs. 15.8%, respectively; P < 0.001 and 10.6% vs. 1.4%, respectively; P < 0.001). A positive LN rate was detected in 66.3% of IDCs and 65.1% of ACCs (P = 0.019). Similarly, tumor metastasis was observed in 13.5% of IDCs and 1.4% of ACCs (P < 0.001). Treatments administered were different between these two groups. The radical prostatectomy rate was lower in patients with IDC than in patients with ACC (46.4% vs. 75.3%, respectively; P < 0.001), and adjuvant radiation was used more frequently to treat IDC than ACC (26.8% vs. 14.8%, respectively; P = 0.001).

**Table 1:** Characteristics of patients with IDC compared to ACC

		IDC, n=511 (%)		ACC, n=3303 (%)		Total, n=3814 (%)		P-Value^a^
Median follow-up (months) (IQR)		38 (17-68)		98 (83-109)		54 (94-107)		
Year of diagnosis	2004-2008	216	42.3	2880	87.2	3096	81.2	<0.001
	2009-2013	295	57.7	423	12.8	718	18.8	
Age^b^	15-54 years	54	10.6	631	19.1	685	18.0	<0.001
	55-64 years	145	28.4	1391	42.1	1536	40.3	
	65-74 years	168	32.9	937	28.4	1105	29.0	
	75-84 years	106	20.7	290	8.8	396	10.4	
	85+ years	38	7.4	54	1.6	92	2.4	
Race	White	378	74.0	2682	81.2	3060	80.2	<0.001
	API	51	10.0	103	3.1	154	4.0	
	Black	73	14.3	457	13.8	530	13.9	
	Unknown	9	1.8	61	1.8	70	1.8	
Marital status	Not married^c^	111	21.7	606	18.3	717	18.8	0.014
	Married	349	68.3	2452	74.2	2801	73.4	
	Unknown	51	10.0	245	7.4	296	7.8	
Grade	Grade I and II	71	13.9	1327	40.2	1398	36.7	<0.001
	Grade III and IV	366	71.6	1922	58.2	2288	60.0	
	Unknown	74	14.5	54	1.6	128	3.4	
Tumor size (cm)	< 2	56	11.0	499	15.1	555	14.6	<0.001
	2-5	56	11.0	173	5.2	229	6.0	
	> 5	6	1.2	15	0.5	21	0.6	
	Unknown	393	76.9	2616	79.2	3009	78.9	
AJCC Stage	I and II	269	52.6	2596	78.6	2865	75.1	<0.001
	III	104	20.4	468	14.2	572	15.0	
	IV	114	22.3	131	4.0	245	6.4	
	Unknown	24	4.7	108	3.3	132	3.5	
AJCC T stage	T1	103	20.2	446	13.5	549	14.4	<0.001
	T2	213	41.7	2256	68.3	2469	64.7	
	T3	115	22.5	522	15.8	637	16.7	
	T4	54	10.6	47	1.4	101	2.6	
	TX	26	5.1	32	1.0	58	1.5	
Surgery type	None	165	32.3	671	20.3	836	21.9	<0.001
	RP	237	46.4	2488	75.3	2725	71.4	
	Unknown	109	21.3	144	4.4	253	6.6	
Radiation	None	361	70.6	2757	83.5	3118	81.8	
	Yes	137	26.8	490	14.8	627	16.4	
	Unknown	13	2.5	56	1.7	69	1.8	
Gleason score	5-7	79	15.5	339	10.3	418	11.0	<0.001
	8-10	130	25.4	42	1.3	172	4.5	
	Unknown	302	59.1	2922	88.5	3224	84.5	
LN status	Negtive	165	32.3	1140	34.5	1305	34.2	0.019
	Positive	339	66.3	2149	65.1	2488	65.2	
	Unknown	7	1.4	14	0.4	21	0.6	
Metastasis at diagnosis	No	426	83.4	3194	96.7	3620	94.9	<0.001
	Yes	69	13.5	46	1.4	115	3.0	
	Unknown	16	3.1	63	1.9	79	2.1	

API = Asian or Pacific Islander, AJCC = American Joint Committee on Cancer, IDC = infiltrating duct carcinoma, ACC = acinar cell carcinoma, IQR = interquartile range, LN = lymph node, RP = radical prostatectomy.

a. P-value (Chi-square test) to compare the IPC and IDC groups.

b. Age Standard for Survival PCa (15-54,55-64,65-74,75-84,>85).

c. Including divorced, separated, single (never married) and widowed.

### Comparison of survival between IDCs and ACCs

**Figure 1:**
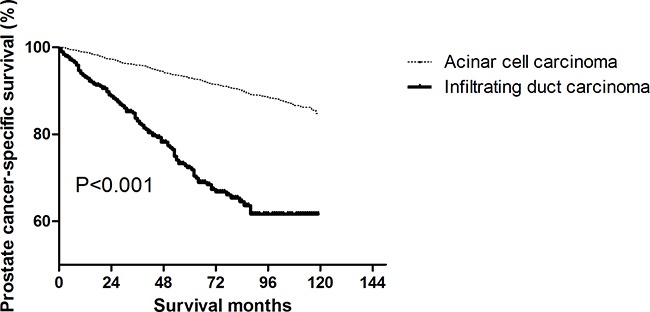
Log-rank test for prostate cancer-specific survival (PCSS) to compare infiltrating ductal carcinoma (IDC) and acinar cell carcinoma (ACC) (P < 0.001)

As shown in Kaplan–Meier plots, PCa-specific survival (PCSS) was worse in patients with IDC than in patients with ACC (P < 0.001, Figure 1). The five-year PCSS rates in IDC and ACC were 72.32% and 92.98%, respectively (P < 0.001). A Cox regression analysis was used to explore the effects of baseline characteristics on PCSS (Table 2). In the univariate analysis, some prognostic indicators were found to be significantly associated with PCSS. These included the year of diagnosis, age, marital status, tumor size, AJCC stage, AJCC T stage, tumor grade, surgery type, radiation, Gleason score, LN status, and tumor metastases (Table 2). Thus, all of these variables were included in the multivariate analysis to confirm the prognostic value of the significant factor identified in the univariate analysis (Table 2). However, after adjusting for other prognostic factors and performing the multivariate analysis, year of diagnosis, marital status, tumor size, AJCC stage, radiation, Gleason score, and LN status were no longer significant independent prognostic factors (Table 2).

**Table 2:** Univariate and Multivariate Analysis of PCa-specific survival (PCSS)

Variables		Multivariate analysis		Multivariate analysis	
		HRs (95% CI)	P	HRs (95% CI)	P
Year of diagnosis	2004-2008	1	-	1	
	2009-2013	2.65 (1.75, 4.01)	<0.001	0.57 (0.28, 1.17)	0.127
Age at diagnosis (years)	15-54 years	1	-	1	-
	55-64 years	0.68 (0.38, 1.22)	0.196	0.67 (0.36, 1.23)	0.198
	65-74 years	1.99 (1.18, 3.35)	0.009	1.23 (0.7, 2.13)	0.473
	75-84 years	6.02 (3.56, 10.19)	<0.001	1.14 (0.62, 2.1)	0.671
	85+ years	20.77 (11.37, 37.94)	<0.001	3.85 (1.86, 7.94)	<0.001
Race	White	1	-	1	-
	API	1.16 (0.57, 2.37)	0.680	0.61 (0.28, 1.34)	0.220
	Black	1.15 (0.77, 1.73)	0.489	1.12 (0.72, 1.75)	0.608
	Unknown	0.65 (0.16, 2.61)	0.539	0.52 (0.12, 2.15)	0.365
Marital status	Not married	1.000	-	1	-
	Married	0.45 (0.32, 0.61)	<0.001	0.9 (0.63, 1.27)	0.538
	Unknown	0.54 (0.3, 0.99)	0.046	0.7 (0.37, 1.31)	0.264
Grade	Grade I and II	1	-	1	-
	Grade III and IV	3.58 (2.34, 5.47)	<0.001	2.47 (1.56, 3.91)	<0.001
	Unknown	12.22 (6.5, 22.98)	<0.001	1.12 (0.54, 2.34)	0.757
Histology type	IDC	1	-	1	-
	ACC	0.11 (0.08, 0.15)	<0.001	0.52 (0.35, 0.78)	0.001
Tumor size (cm)	< 2	1	-	1	-
	2-5	2.45 (0.89, 6.75)	0.084	1.66 (0.59, 4.66)	0.336
	> 5	18.56 (5.59, 61.65)	<0.001	3.44 (0.84, 14)	0.085
	Unknown	4.06 (2, 8.26)	<0.001	1.1 (0.52, 2.31)	0.799
AJCC Stage	I and II	1	-	1	-
	III	2.21 (1.38, 3.54)	0.001	1.34 (0.55, 3.27)	0.519
	IV	28.82 (20.63, 40.27)	<0.001	1.65 (0.73, 3.74)	0.228
	Unknown	4.04 (2, 8.15)	<0.001	1.51 (0.47, 4.82)	0.487
AJCC T stage	T1	1	-	1	-
	T2	0.28 (0.19, 0.42)	<0.001	1.16 (0.75, 1.79)	0.510
	T3	0.73 (0.47, 1.15)	0.180	2.87 (1.36, 6.05)	0.006
	T4	5.4 (3.36, 8.68)	<0.001	2.74 (1.42, 5.27)	0.003
	TX	6.51 (3.62, 11.69)	<0.001	1.65 (0.82, 3.28)	0.158
Surgery type	None	1	-	1	-
	RP	0.09 (0.06, 0.13)	<0.001	0.1 (0.05, 0.19)	<0.001
	Unknown	2.1 (1.48, 2.98)	<0.001	1.1 (0.74, 1.66)	0.631
Radiation	None	1		1	-
	Yes	1.53 (1.08, 2.17)	0.017	0.69 (0.44, 1.07)	0.099
	Unknown	1.25 (0.4, 3.91)	0.706	0.98 (0.3, 3.24)	0.980
Gleason score	5-7	1		1	-
	8-10	21.25 (6.31, 71.56)	<0.001	1.75 (0.47, 6.45)	0.401
	Unknown	2.58 (0.82, 8.13)	0.106	0.88 (0.24, 3.31)	0.854
LN status	Negtive	1		1	-
	Positive	3.53 (2.29, 5.43)	<0.001	0.95 (0.53, 1.67)	0.850
	Unknown	10.83 (2.55, 45.88)	0.001	2.13 (0.38, 11.84)	0.388
Metastasis at diagnosis	No	1		1	-
	Yes	62.22 (45.18, 85.68)	<0.001	7.84 (3.79, 16.22)	<0.001
	Unknown	3.81 (1.77, 8.21)	0.001	1.09 (0.31, 3.84)	0.890

a. CI = confidence interval, HRs = hazard ratios, IDC = infiltrating duct carcinoma, ACC = acinar cell carcinoma, API = Asian or Pacific Islander, AJCC = American Joint Committee on Cancer, LN = lymph node.

b. Not married including divorced, separated, single (never married) and widowed.

### Subgroup analyses

**Figure 2:**
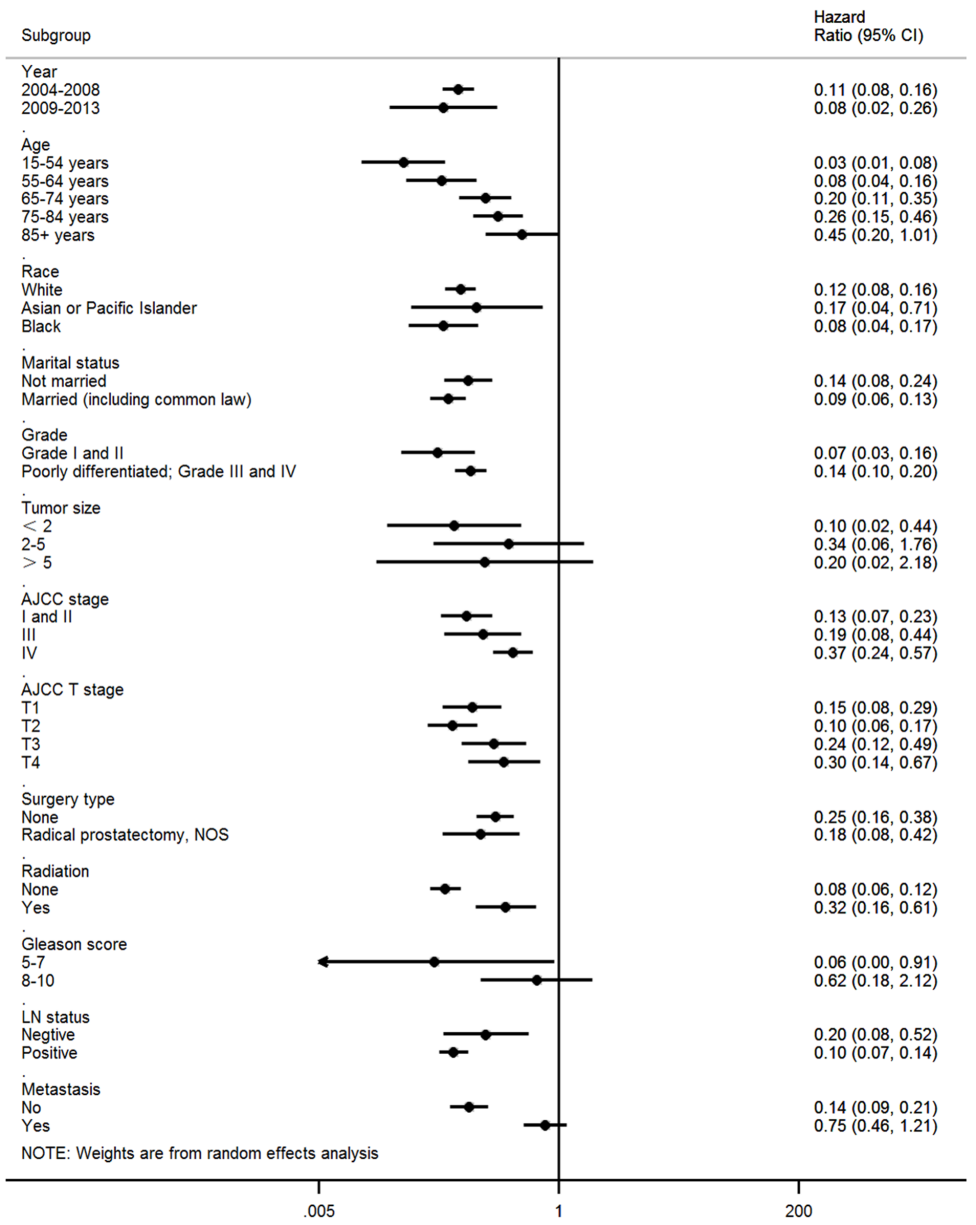
Forest plot of hazard ratios (HRs) comparing the subgroup analysis of predictive values of prognosis factors for infiltrating ductal carcinoma (IDC) versus acinar cell carcinoma (ACC)

A forest plot of hazard ratios (HRs) was used to illustrate the exploratory subgroup analyses (Figure 2). HRs in the subgroup of patients over 85 years of age were not significantly different between the two arms (HR = 0.45, 95% confidence interval [CI] 0.20–1.01; P = 0.053). Similarly, HRs in the subgroup of patients with larger tumor size were not significantly different between the two arms (tumor size>5: HR = 0.20, 95% CI 0.02–2.18, P = 0.085). HRs in subgroups with Gleason score 8–10 were not significantly different between the two arms (Gleason score 8-10: HR = 0.62, 95% CI 0.18–2.12; P = 0.446). HRs in subgroups with metastasis at diagnosis were not significantly different between the two arms (HR = 0.75, 95% CI 0.46–1.21; P = 0.235).

## DISCUSSION

To the best of our knowledge, only Meeks et al. [4] has comprehensively described the features of IDC. However, the study Meeks et al. did not analyze some important demographic and clinicopathological characteristics of patients, including marital status, grade, tumor size, AJCC stages, and Gleason score. It is particularly noteworthy that this is the first study to evaluate the characteristics of Asian and Pacific Islander men with PCa. In multivariate Cox regression analysis, Asian and Pacific Islander ethnicities were not independent prognostic factors of IDC. To illustrate the subgroup analyses, we generated a forest plot. This graph showed a superior prognostic value for IDC among individuals of Asian or Pacific Islander ethnicity. Additionally, this study is the first to report the clinicopathological features and survival outcomes of patients over 85 years of age. In multivariate Cox regression analysis, age over 85 years was an independent prognostic factor. However, in the subgroup analyses, age over 85 years did not have a superior prognostic value for IDC.

The dramatic differences in incidences and outcomes between patients with IDC and those with ACC were highlightedin this analysis of SEER data. To date, the incidences and numbers of patients with IDC remain unknown. Therefore, it is desirable to obtain more information with respect to the clinical and pathological features of IDC.Thus, we conducted a study on a large population to gain a sufficient number of patients with those relatively rare subtypes of PCa.

To date, our research represents the most comprehensive study of IDC, including the most complete description of tumor and clinicopathological characteristics. Our results showed that IDC has unique clinicopathological characteristics. After summarizing the tumor and clinicopathological characteristics of IDC, we found that IDC was associated with a higher grade, a larger tumor size, increased LN involvement, latter AJCC stages, higher AJCC T stages, less radical prostatectomy rate, higher radiation rate and higher tumor metastasis rate. Some of these results are in agreement with those of previous reports [4–7]. In univariate analysis, survival was reported to be significantly worse in patients with IDC than those with ACC, which is consistent with previous studies [4, 5, 8]. Collectively, the results of a multivariate Cox regression analysis showed that the survival was also worse in the IDC group. Further, these results showed that the IDC histological type itself is an independent prognostic factor. A subgroup analysis was performed to identify the underlying factors that contributed to this phenomenon. Interestingly, the outcomes of the subgroup analyses indicated that the Gleason score had no superior prognostic value for IDC.

The outcomes of this research have several therapeutic implications. We found that the histological type was an independent prognostic factor in multivariate analysis. This finding indicates thatfor IDC management should be individualized and tailored. IDC is a rare subtype of PCa with poor prognosis. [9] In terms of worse survival outcomes of IDC, timely measures are needed to successfully manage patients with this rare subtype. Moreover, both the multivariate and subgroup analyses indicated that the Gleason score is not a prognostic factor for IDC. Although the Gleason score is commonly used to evaluate the biological behavior of PCa, cliniciansmay need to take into account other prognostic factors rather than the Gleason score in order to accurately evaluate patients with the IDC subtype.

There are several limitations to this study. First, the SEER database did not include prostate-specific antigen records until 2010, which precluded us from obtaining potentially relevant prognostic factor data on PSA. Second, we enrolled patients who were diagnosed with PCa between 2004 and 2013 as large amounts of useful data including tumor size, Gleason score, LN status and metastasis at diagnosis on PCa were not available before 2004. This may have resulted in bias, and thus, could decrease the external validity of our results [10].

## CONCLUSION

The IDC subtype of PCa has unique clinic-opathological characteristics and is associated with a worse prognosis compared with ACC. Multivariable Cox regression analysis showed that age over 85 years, grades III and IV, T3 and T4, and metastasis at diagnosis were identified as independent prognostic factors of worse survival outcomes, whereas radical prostatectomywas found to be an independent prognostic factor of better survival outcomes. However, in subgroup analyses, age over 85 years and metastasis at diagnosis were no longer significant predictors of worse survival outcomes. Further validation of these results in a large population may help to clarify this issue. Improving our understanding of the clinical and biological features of IDC may offer more individualized and tailored therapies for PCa patients.

## MATERIALS AND METHODS

### Ethics statement

Permission was obtained to access the SEER research data files using the reference number 12256-Nov2015. The data released by the SEER database do not require informed patient consent, and our study was approved by the Ethical Committee and Institutional Review Board of The First Affiliated Hospital of Fujian Medical University. The methods were performed in accordance with the approved guidelines.

### Data acquisition and patient selection

We used the SEER dataset that was released in April 2015. This dataset included data from 18 population-based registries (1973–2013). Data for tumor characteristics were recorded based on the International Classification of Diseases for Oncology Version 3 (ICD-O-3). The patient inclusion criteria were as follows: male sex, over 15 years of age, first and only cancer diagnosis of PCa, prostate gland was the only primary site, pathological confirmation of IDC (ICD-O-3 8500/3) and ACC (ICD-O-3 8550/3), surgical type, American Joint Committee on Cancer (AJCC) stages I–IV, and diagnosis between 2004 and 2013. Patients diagnosed with PCa before 2004 were excluded because the SEER database was not available for some important tumor characteristics, including tumor size, derived AJCC stage group (6^th^), derived AJCC T (6^th^), Gleason score and metastasis at diagnosis until 2004. Additionally, patients diagnosed with PCa after 2013 were excluded as the SEER database was available before 2013 and adequate follow-up time could not be ensured. Patients diagnosed with PCa based on a death certificate or autopsy was also excluded. A total 3814 patients were enrolled in this study. Among these patients, 511 were diagnosed with IDC and 3033 were diagnosed with ACC.

The collected demographic statistics included the year of diagnosis, race, age, and marital status. Age was classified using the age standards for survival for PCa: 15-54, 55-64, 65-74, 75-84 and >85 years of age. Histologic grade, AJCC stage, AJCC T stage, tumor size, surgery type, radiation, Gleason score, regional LN status and metastasis at diagnosis were treated and included as tumor characteristics. Among these variables, tumor size was classified as follows: < 2 cm, 2 to 5 cm, and > 5 cm.

### Outcome measurements

In this study, PCSS was treated as the primary study outcome. It was calculated from the date of diagnosis to the date of death secondary to PCa. Patients who died from other causes unrelated to a PCa diagnosis or who were alive were censored on the date of death or the date of last contact. The cut-off date was predetermined by SEER 2015 submission databases, which contained data of deaths until 2013. Thus, December 31, 2013 was the study cut-off date.

### Statistical analysis

Clinicopathological features were compared across groups using Pearson's Chi-square tests, Fisher's exact tests, Cochran-Mantel Haenszel, or Chi-square tests. The Kaplan-Meier method was used to generate survival curves. Log-rank tests were used to analyze the differences between curves. Univariate and multivariate Cox hazard analyses were used to determine factors that are correlated with PCSS, and HRs and 95% CIs were reported. Subgroup analyses were performed to evaluated the HRs of IDC versus ACC. In order to compare the predictive value of each prognostic factor on PCSS, we generated a forest plot. SPSS version 22.0 (IBM Corp, Armonk, NY) was used to perform statistical analyses. Kaplan-Meier curve was plotted with GraphPad Prism version 5 (GraphPad Software, Inc., La Jolla, CA, USA). A two-sided P < 0.05 was considered to indicate statistical significance [11].

## Abbreviations

SEER: the Surveillance, Epidemiology, and End Results
IDC: infiltrating ductal carcinoma
ACC: acinar cell carcinoma
PCSS: PCa-specific survival
HR: hazard ratio
LN: lymph node
AJCC: American Joint Committee on Cancer
Acquisition of data: Shao-Hao Chen, Shi-Tao Wang, Xiao-Dong Li, Hai Cai, Yun-Zhi Lin, Xue-Yi Xue, Yong Wei, Qing-Shui Zheng
Analysis and interpretation of data: Yu-Peng Wu, Shao-Hao Chen, Shi-Tao Wang
Drafting of manuscript: Yu-Peng Wu, Shao-Hao Chen, Shi-Tao Wang, Ning Xu.
Critical revision: Yu-Peng Wu and Ning Xu.
